# 

**Published:** 1859-10

**Authors:** 


PUBLISHED MONTHLY, AT $3 PER ANNUM IN ADVANCE.

### ATLANTA

### MEDICAL AND SURGICAL

### JOURNAL.

EDITED BY

JOSEPH P. LOGAN, M. D.,

Professor of Physiology and Diseases of Women and Children in the Atlanta Medical College.

AND

W. F. WESTMORELAND, M. D.,

Professor of the Principles and Practice of Surgery in the Atlanta Medical College.

Pax et scientia, sed veritas sine timore.

Vol. V. OCTOBER, 1859. No. II.

ATLANTA, GEORGIA:

PRINTED BY J. I. MILLER & CO.

(Successors to G. P. Eddy & Co.)

1859.

EACH NUMBER CONTAINS SIXTY-FOUR PAGES.

